# The Dental Society of the State of New York

**Published:** 1896-12

**Authors:** 


ARTICLE VII.

### THE DENTAL SOCIETY OF THE STATE OF NEW YORK.

The twenty-eighth annual meeting of the Dental Society of the State of New York was held at Albany on May 13, 14, 1896, and the attendance was largely in excess of that of recent years, an evidence that interest in Society work is by no means dead, but dependent merely upon the attractiveness of the programme offered.

Dr. H. J. Burkhart, of Batavia, the President, delivered a very able and interesting address, in which he touched upon many points of vital interest to the members, and suggested several reforms in Society management. These were referred to a special committee, who finally recommended their adoption, and they will be enforced during the current year. As Dr. Burkhart was unanimously re-elected, he will be in the position to inaugurate the various changes, and their value will thus be tested.

The correspondent's report was of unusual interest and is of extreme importance, because it is mainly a compilation of the opinion of American dentists practicing in Europe upon the practicability of utilizing the vitreous materials with which the filling of teeth has been attempted. In European countries there is a deep-seated repugnance to gold in conspicuous places, and there is necessarily a corresponding effort on the part of dentists to supply the demand for a filling which matches tooth substance. The report follows:

### REPORT OF CORRESPONDENT.

*Mr. President and Members of the State Society:*

It is the duty of your correspondent to gather the opinions of practicing dentists upon some matter of interest to the profession at large, and to report at this meeting, This year it occurred to me that, wh reas American dentistry claims ascendency over the practice in other parts of the world, there is one method upon which we might receive information, if not instruction, from our brothers across the water.

In this country we have educated our patients to the point where they no longer look upon gold, in the front of the mouth, as a disfigurement, but either as a necessary evil, bearable because all others endure the same infliction, or in many cases as a positive adornment. In this latter respect I regret to say that many unscrupulous men unhesitatingly crown incisor teeth with solid gold crowns, telling confiding and vain patients that it is the fashion; or in some instances they have even placed gold crowns in which they have inserted flashing diamonds, so that what was looked upon as a myth in dental practice is now at last a common sight in the metropolis. I recently saw a woman (evidently not a lady), in whose mouth four gold crowns conspicuously showed, two of which were set with diamonds. This is a practice as degrading to the science of dentistry as it is meretricious on the part of the patients.

In Europe a contrary state of affairs exists. The plastic fillings are more in vogue than gold, and hence the dentists of Europe have continued to seek a solution of the porcelain-filling problem, even though such a method could not promise so much as gold. They hope to do better with it than with phosphate or gutta-percha alone.

Recently, I have been led to believe that the Europeans have brought this practice to a high state of perfection, and therefore I essayed to obtain a report of the status of that method of practice.

I addressed the following letter to twenty-five leading practitioners, a few of whom have obligingly replied:

"As correspondent of our State Society, it becomes my duty to report each year upon some important advance in dental science. I have learned that porcelain fillings are much more in vogue on your side of the water than in this country, and therefore I have decided, this year, to ask the leading men among our European *confreres* for an expression of opinion upon this subject. Will you let me have a statement of the results of your experience with porcelain as a filling material? And will you cover the following points? What is your method? To what class of cavities do you limit its use? What cement do you use? How long do the fillings remain in position? Is it a satisfactory substitute of gold, aside from esthetic considerations?"

Dr. William Slocum Davenport, 30 Avenue de l'Opera, Paris, sends the following:

"I have found where it is possible to secure an accurate impression of a cavity, that the operation can be made a perfect success, and is a satisfactory substitute for gold aside from esthetic considerations. For large cavities, soft and frail teeth, and for nervous or elderly patients, it is especially useful.

"The impression is obtained by pressing No. 40 crystal (rolled?) gold into the cavity with small pieces of amadon. The gold is burnished to the cavity, and over its extreme edge. It is then removed by inserting a sharp explorer point into gold at the bottom of the cavity and lifting gently (should the gold be punctured it is immaterial), and invested in a paste of powdered asbestos and water.

"With the impression in this position we remove excess of water with blotting paper, and are then able to fill it about half full of the glass material and bake at once under flame of the blow-pipe, in Cunningham's, Downie's or any other furnace. Two or three bakings are usually necessary. Cooling after baking is obtained in a few seconds by placing the bottom of the spoon in a few drops of water. The investing and baking require but a few minutes. Try the filling in the cavity first with the gold intact.

If necessary, grind off excess of contour and burnish gold at the edge of the filling, which will at the same time break all feather edges from the filling.

"Remove, invest, and heat until the surface takes a polish. This is very important.

"In building up cusps or contour surfaces cover the portion of the filling which is not to be changed, with investing material, then add porcelain as desired.

"The success in this artistic branch of dentistry is in proportion to the perfection of the operation. Fillings which I finished accurately about four years ago are still in every way good. When I relied on cement for badly fitting fillings black and rough edges have resulted, and fillings which required grinding and polishing after cementing have turned dark gray and sometimes nearly black.

"F. Meyer's glass preparation is far superior to any I have used. It flows to the surface of the gold as though it were solder. It makes a hard filling which has a surface like that of an English tooth. Materials made by Cunningham's, Downie's, and Reisert's, also Allen's gum enamel, are most valuable in producing gum color, green-stain, or tobacco teeth. George Poulson's plumbago granite cement is no doubt very good for setting inlays, but undercuts in cavities and grooves in the filling are very necessary. Porcelain filling occupies a position apart, which no other material can fill."

Dr. Isaac B. Davenport writes as follows:

"Obtain an accurate impression of the cavity, No. 49 gold foil, extending the gold over the edge as a guide to contour. Place a portion of the material in the impression, and fuse over the spirit lamp or Bunsen burner. If there is any doubt of the perfection of the impression, try it in with first layer of fused porcelain, and if it rocks in the cavity, the porcelain is broken down and the gold pressed against the wall. Remove, add new material, and fuse again. This method of repeated fusing overcomes the effects of contraction.

"When the filling is perfect in color and form, the gold is peeled off, retention grooves cut with a diamond disk, and it is ready to be set with any cement suitable for setting crowns. The cement should approach the color of the inlay.

"In large or difficult fillings I employ the method described by Dr. W. S. Davenport, investing in the powdered asbestos and water.

"The filling should fit perfectly without grinding. I always fear that a ground surface will absorb organic matter and become discolored.

"The fusing should be perfect each time, but overheating destroys the color. I usually select a shade darker than seems necessary.

"I have fillings which have stood six to eight years; but in some mouths the thin line of cement dissolves out in a year or two and needs replacing. A well-filled porcelain filling set in cement will last much longer than a cement filling in the same mouth.

"I prefer porcelain to gold solely for esthetic reasons, and only use it on visible surfaces. I am not convinced that it is a fit material for large compound cavities in bicuspids or molars, unless perhaps in a tooth standing alone.

"Laterly I have set inlays with gutta-percha and am inclined to think this may prove better than cement."

Dr. A. V. Elliot, 10 Via Tornabuoni, Florence, Italy, sends the following:

"After several years' experience I find that for certain cavities this method is eminently satisfactory, especially where the appearance of gold would be unsightly.

"I prepare the cavities as for gold, then fill the undercuts with wax to facilitate the removal of the matrix.

"I use Myer's prepared powder, and form the matrix with No. 60 or 80 gold foil, or gold and platinum, the gold being on one side and platinum on the other. The gold is pressed into the cavity with a small, stiff wad of cotton and afterward burnished. I use Harvard cement, because of its adhesive and other good qualities.

"Though not as permanent as gold, porcelain fillings are more durable than either white fillings or gutta-percha alone, and certainly far more artistic.

"It must be remembered, also, that the ladies of thts country, not having reached so high a degree of barbaric civilization as the Americans, are unwilling to have gold pounded into their teeth where it can be seen. Hence, under the law of least resistance, we are under a greater necessity to disguise our work."

Dr. Charles J. Monk, 12 Wilhelmstrasse, Wiesbaden, Germany, writes as follows:

"From my own experience and that of my colleagues whose work I have seen, I am satisfied that porcelain fillings should have a place in the repertoire of every dentist. Where the porcelain is accurately adapted, these fillings I think must be as durable as gold.

"My method is as follows: I obtain the small buttons of porcelain which Ash & Sons supply in various forms and shades, fit as well as possible and set with carbon cement, mixed thin, grinding and polishing the next day.

"The durability of porcelain fillings depends upon the close joint obtained, so that little or no cement is exposed.

"In the three years during which I have done this work, I have had none come out except in approximal cavities where the facing is porcelain and the lingual surface cement. With these several cases the cement has washed away, porcelain remaining in position, and I have simply added more cement."

Dr. Waldo E. Royce, 2 Lonsdale Gardens, Tunbridge Wells, England, sends me the following:

"English patients strongly object to conspicuous gold fillings. The dental profession has therefore been forced to look for some substitute possibly less durable but more esthetic. Of all these substitutes whether considered from the point of durability or appearance, I am convinced that porcelain stands first.

"My first operations of this kind were performed about fourteen years ago. I then ground pieces of Ash's teeth to fit the cavity. These operations have proven most satisfactory both to patient and operator, but the time consumed and the consequent fee placed them beyond the reach of most patients. Mr. Dall, of Glasgow, has amplified this method, and at the last meeting of the American Dental Society of Europe showed some very beautiful specimens.

"I next used Richard's glass fillings, and for small cavities I find them reliable. I have made dozens of them, and it takes a sharp eye to detect some that have already stood for about five years. In the mouths of my patients small glass fillings do not change color. I have not been so successful with large ones. I set with Harvard cement, the rough surface of the glass giving a particularly strong hold.

"The cement should also be of a color to match the tooth, and the filling covered like a cement filling with sticky wax. These fillings can be made without leaving the chair, and in less time than is usually required for a gold filling.

"Of the porcelain, I have attained the best results with a body which comes from Dresden. It is worked very much like the glass, but it requires such a high temperature to fuse it that we are instructed to invest the matrix in powdered asbestos, holding in a platinum spoon and fusing with a blow-pipe.

"This method I have found very uncertain on account of the danger of smoking the inlay. I have of late used Downie's furnace with good results. Much should be done by the manufacturers of all these bodies in the way of improving the shades. It is absolutely necessary both for appearance and durability that the porcelain should perfectly fit the cavity. It should therefore only be used in cavities which are easy of access. For such cavities I think it is the coming material, but with our present knowledge of its manipulations it should not be used as a substitute for gold except where its esthetic qualities are essential."

Dr. A. T. Webb, 87 Via Nazionale, Italy, writes as follows:

"I have been using porcelain for inlays and fillings during a period of about four years; at first grinding in pieces, and later adopting the system of burnishing platinum foil to the cavity and building up with bodies. Naturally at first I confined such work to the anterior teeth and buccal surfaces, with considerable satisfaction; but later, with the confidence engendered by closer adaptation, I extended it to include all classes of cavities, and with results which have exceeded my anticipations.

I have used it in all classes of teeth, both from esthetic and conservative principles. In cases where the showing of gold was unavoidable, in the anterior approximal cavities of the upper bicuspids, I have used the porcelain with so far good results. Some which I did two and a half years ago are in excellent condition, and bid fair to endure for several years. In those teeth where the plastics are strongly indicated, I have found the porcelain of considerable value. By its use the life of an ordinary cement filling is much prolonged.

"From my own experience, and observation of the work done by colleagues, I should say that three years would be a fair average of durability. As to being a satisfactory substitute for gold in select cases, Yes. But for all-around use gold must be awarded the premium as heretofore."

Dr. E. A. Galbreath, Hanover, writes as follows:

"I include under the head of porcelain fillings also those made of glass. I use them only where esthetically anything else would be impossible, as in incisors, cuspids, etc. For restoring corners, I use glass, fusing in the wellknown way on platinum-gold foil. For cavities not extending as far as the cutting edge I use Ash's right and left porcelain rods. They give me great satisfaction. I grind them in—they fit fairly well already—cut them off, set in cement, and then polish. I use Poulson's cement.

"Small glass contour fillings I reset every two years. The cement should be mixed very thin, or the block breaks in setting. Large blocks hold much longer, because the cement can be mixed harder. Of course all cements dissolve, other things being equal, according to the amount of powder incorporated with the crystals (liquid). The durability of porcelain fillings is limited by the durability of the very perishable cement with which they are set, so that in this respect there is no comparison between them and gold fillings. They give, however, my patients and myself so much satisfaction that I am using them more all the time,"

Dr. Wilhelm Sachs, Tayentzjenstrasse 3a Breslau, writes as follows:

"I prepare the cavity, leaving sharp edges and no undercuts. I then take an impression of the cavity with Stent's impression material, and make a model with cement filling material. After hardening, I grind a piece of artificial tooth, or, better, a porcelain inlay from S. S. White, suitable in shade, to fit the cavity of the cement model, grinding it a little conical until it will go about half way into the cavity. I then fasten the porcelain piece with shellac to an old broken excavator, and fit it as exactly as possible into the cavity of the tooth."

"I use porcelain for labial surfaces of incisors, cuspids, and first bicuspids; occasionally for mesial surfaces of second bicuspids if first bicuspid is lost. I never use it for restoring a corner of incisors or cuspids when the pulp is alive. I have had many such failures, although in some cases corners will stand for more than ten years. But these are exceptions. When the pulp is dead, I restore the lost corner by taking an artificial tooth, soldering a gold post to the pins, and fitting it to the tooth.

"I use Weston's cement, mixed thin; sometimes Poulson's cement; but this, although it adheres very well to the walls of the cavity, is too sticky, and if not missed very thin it does not harden thoroughly.

"After porcelain is inserted I leave the dam in place for some time, but grind and polish next day. If you attempt it in the same sitting, often the porcelain will jump out.

"I have a large number of porcelain inlays which have stood now over ten years; but in all cases the porcelain must be surrounded by tooth substance.

"Porcelain fillings in the right place are a very satisfactory substitute for gold; for esthetic purposes porcelain is the best of all filling-materials;

"I use glass fillings, sometimes taking an impression of the cavity with gold-platinum foil in which I fuse the inlay.

"The glass filling must never extend over the edge of the cavity. If you grind surplus off, you get a porous surface which will discolor in some mouths. Glass fillings are very satisfactory, if you make them so perfectly that the upper glossy surface is just level with the tooth surface, so that no grinding at all is required. If the glass filling is properly done, it will stand a long time. I have about twenty cases which I have watched for about seven years, and they are in good order. The glass filling must be fastened with very thin Weston's cement.

"I do not think glass and porcelain fillings are used more extensively in Germany than in America. Only few first-class dentists will take the trouble to make glass or porcelain fillings, as the time required for it is about four or five times as great as for a gold filling."

Dr. William Mitchell, 39 Upper Brook street, London, W., England, writes as follows:

"Personally I have had comparatively but little experience in that class of operations, for the reason that it has not appealed to me as a reasonably perfect operation in the majority of cases where undertaken; this conclusion being arrived at from personal experience, but more especially from observations of operations of others who demonstrated by their attempts that they had had considerable practical experience with this line of operations.

"As a rule, I have found the inlay method the most successful in my hands—i. e., selecting a piece of porcelain of the desired shade and grinding this to shape; this, when set in position, can be contoured and finished flush with the tooth, and given the requisite degree of polish. In this connection I would say English teeth are the best adapted to this class of work, owing to their uniformity of texture and the way they can be polished after the vitreous surface is removed. I would like to mention here the enamel-rods of various shades and sizes devised and prepared under the instructions of Mr. Dall, of Glasgow who has devoted a great deal of time and personal effort to perfect a system for this kind of work, and who deserves the heartiest thanks from our profession for his untiring energy in this direction.

"I have done but little with porcelain fillings, and the best results in my hands as to shades and shapes leave much to be desired. While it is possible to approximate the desired color, the least fluctuation of heat will spoil an otherwise fair operation. I say fair advisedly, for I have but rarely seen a good one—i. e., one that I would wish in my own mouth or consider a specimen operation, considered in all its points as compared with a gold filling.

"This has special reference to porcelain fillings as distinguished from inlays. The class of cavities to which I have confined my efforts are erosions, abrasions, and carious cavities upon the labial surfaces of the anterior teeth. The operation as to the two former is practically alike. The cavity is prepared very much the same as for a gold filling, except that the margins are left as nearly perpendicular as possible—i. e., at right angles to the floor of the cavity, instead of beveled as in a gold filling. The piece of porcelain is then ground to fit. This requires an amount of precision rarely given to this operation, for upon the degree of approximation depends the result. The eroded or abraded cavities are irregular in shape, and frequently require much time in the operation of restoring lost tissue, but the results will repay all time and care bestowed in this direction.

"A method used by my brother, Dr. L. J. Mitchell, possesses many advantages, although the operation involves much time and considerable ability to produce the best results. The process is as follows: Given a case where the loss of tissue involves nearly the whole face of a tooth, the edges are to be as well defined as possible; the incisive end of the cavity is to be undercut by running an inverted cone bur transversely in the cavity, while the cervical portion is to be prepared as for a gold filling. The piece of porcelain is then fitted with the incisive and beveled to fit in the undercut, while the cervical portion is beveled in like manner. After the filling is perfected, and after applying the rubber-dam, the piece of porcelain is cemented in place. After the cement has become perfectly hard, that along the cervical margin is cut away from the retaining groove, as well as any that may be along the cervical bevel of the piece of porcelain. The retaining groove is then filled with gold, which is carried over the bevel on the porcelain; the whole is then harmoniously contoured to the required shape and polished. The inlay is then held in place most effectually, and an extremely neat operation is the result.

"When a very thin piece of porcelain is used and cement alone depended upon for its retention, it is very prone to loosen, owing to the porosity of the porcelain being accentuated through the removal of the vitreous surface during its preparation, or its subsequent grinding down when *in situ*. This permits of the oral fluids penetrating it, and disintegrates its union with the cement, which eventuates in the early failure of most of this work.

When possible, as in the case of caries on the labial surfaces of the teeth, the cavity is made circular, the piece of porcelain is mounted with shellac, or preferably sealing wax, upon one of the metal mandrels suggested by me some years ago (and now obtained at any dental depot.) This after close approximation upon a diamond disk, the mandrel being run in the engine, the piece of porcelain can then be ground into the cavity by the aid of very fine pumice powder and water. This will make an inlay necessitating the minimum amount of cement, which is a benefit in all cases, as I believe the less cement required the better the operation and the more certain the result. Where possible, retaining grooves should be cut along or around the inlay a short distance from the surface. This may be easily done with a diamond disk. This facilitates the adhesion of the cement. I have set a few inlays with gutta-percha, Hill's stopping and Jacob's gutta-percha being about equally used; the latter seems to be the most satisfactory. Any fine grained, quick-setting cement will do for this work, provided the shade approximates as near as possible the color of the natural tooth; otherwise the absence of translucency incidental to this work will be accentuated. Of the comparatively few operations I have done, I am well satisfied as to their durability; the artistic side, I think in most cases, left something to be desired. Esthetic considerations being put aside, I consider a good gold filling far and away the best thing where the patient's personal appreciation of the operation supplements hygienically the ability and devotion shown by the dentist."

Dr. Jenkins, of Dresden, writes that he believes that his most recent discoveries in connection with low-fusing porcelains bring the work to a high state of perfection, but that until he has tested some of the details of his present methods a little longer he prefers not to publish. From what I have heard of the work done by Dr. Jenkins, I am satisfied that his statement is correct, and the method which I have myself followed is his method as it has been described to me.

Before giving my own views on this subject, I wish to call attention to the fact that even in Europe, where porcelain or glass fillings are more in vogue than in this country, the dentists admit that gold is a more permanent reliance, and the esthetic considerations explain the demand for porcelain.

In my hands the method has proven neither so satisfactory nor so simple as its advocates in this country have undertaken to claim for it. While it is true that the porcelain may be fused in a few minutes, it is also true that to make a perfect filling may require an hour or more for a cavity which could readily be filled with gold in half the time. Of course, constant practice, coupled with experience, will give the practitioner an acquired skill which will enable him to fill with porcelain more rapidly than with gold, where the cavity is extensive. I will give a brief outline of the method which I have followed, and record the obstacles with which I have met.

If the process is to be limited to labial surfaces of the anterior teeth, it is evident that the demand for the filling would be comparatively infrequent. Therefore let me consider an approximal and grinding-surface cavity in the anterior of a first bicuspid. The matrix is made with No. 30 rolled gold. This is preferable to 40 or 60, because it takes a sharper impression. To obtain this matrix is most difficult. It is very simple for the advocate of the method to say "make the matrix," and then pass on to the methods of fusing the porcelain. But the dentist will discover that many attempts to obtain a perfect matrix from an approximal cavity will prove abortive before one will be removed which is absolutely accurate, and if not absolutely accurate it is worthless. Practice has taught me that the cavity must be shaped so that, in the class of cavity which I am describing, the matrix may be withdrawn through the opening of the grinding-surface. The gold is folded in half, forming a V-shaped trough, and is passed into the cavity. Then proceed to fill the cavity with small pieces of spunk, holding the pieces in position with a small ball burnisher. No great pressure should be attempted until the cavity is practically filled, otherwise the gold will be torn, which in my experience *does* make a difference, and therefore should be avoided. The gold is burnished over the edges of the cavity with spunk, used with a wiping motion. To remove the matrix, take out all of the spunk, and then "tease" the matrix out. By this expression I mean that the matrix is to be tapped gently along the edges, and manipulated with great care until it drops out of itself. Any forcible removal will alter the shape.

The powder is mixed with water and placed into the matrix, when excess of water is extracted with bibulous paper. For the first baking fill about half full. Fuse thoroughly. Then add material, and fuse again, until the desired shape is obtained. Here occurs a step which is original with Dr. Jenkins. The filling being complete, he lays over it a bit of blown glass, and again fuses, the melted glass flowing down smoothly and forming a glazed or enamel surface of great beauty.

The main difficulty with this method is in connection with the cement. Before cement is used, the filling placed in the cavity is perfection itself, and if the color has been accurately imitated, it would be impossible for human eye to detect the outlines of the filling. But the insertion of the cement changes all this. When it is remembered how thin was the matrix, it will be recognized that no cement now on the market can be mixed thin enough to fill the space occupied by the gold, and consequently the filling is lifted from its proper position by the interposition of the cement, and an edge shows. To overcome this I have essayed the following method: After making the filling, grind away a considerable portion with a corundum, being careful not to touch the extreme edge. This makes space for the cement, and by roughening the surface of the porcelain allows the cement to stick better. Place cement in the cavity, not on the filling, and in a very small quantity. Press the filling to place, and if any cement oozes out, remove the filling, quickly cleanse the edges, and as quickly return it to place. In this manner I have inserted a few fillings which have quite satisfactory edges.

At first, as advised, I used Harvard cement, and I had great difficulty. The fluid is so thick that the mixing to a thin consistence is well-nigh impossible; moreover, the setting is so slow that the fillings would come out in a few days, undoubtedly the fluids of the mouth having disintegrated the cement before fully set.

I have fillings in, which have remained for six or eight months, and which are still doing well, which I inserted with Dawson's cement. The best cement will be that one which is of fine grain, and which sets moderately slowly. Then it is best, after setting the filling, to have the patient remain in the office with dam in place, for half an hour or longer.

At the present stage of this process, I can recommend and even advocate it in accessible positions, where the question of time and fee are of less consideration to the patient than personal appearance. But my own experience, even with the wealthy of this country; leads me to the belief that, ordinarily, Americans will prefer gold.

In accessible places the gold must be our reliance, and I have little faith in porcelain for contouring corners. —*Items of Interest.*

